# Injectable pre-cultured tissue modules catalyze the formation of extensive functional microvasculature in vivo

**DOI:** 10.1038/s41598-020-72576-5

**Published:** 2020-09-23

**Authors:** Nicole E. Friend, Ana Y. Rioja, Yen P. Kong, Jeffrey A. Beamish, Xiaowei Hong, Julia C. Habif, Jonathan R. Bezenah, Cheri X. Deng, Jan P. Stegemann, Andrew J. Putnam

**Affiliations:** 1 Department of Biomedical Engineering, University of Michigan, Ann Arbor, USA; 2 Division of Nephrology, Department of Internal Medicine, University of Michigan, Ann Arbor, USA; 3 Department of Chemical Engineering, University of Michigan, Ann Arbor, USA

**Keywords:** Biomaterials, Cell delivery, Regenerative medicine, Tissue engineering, Cardiovascular diseases

## Abstract

Revascularization of ischemic tissues is a major barrier to restoring tissue function in many pathologies. Delivery of pro-angiogenic factors has shown some benefit, but it is difficult to recapitulate the complex set of factors required to form stable vasculature. Cell-based therapies and pre-vascularized tissues have shown promise, but the former require time for vascular assembly in situ while the latter require invasive surgery to implant vascularized scaffolds. Here, we developed cell-laden fibrin microbeads that can be pre-cultured to form primitive vascular networks within the modular structures. These microbeads can be delivered in a minimally invasive manner and form functional microvasculature in vivo. Microbeads containing endothelial cells and stromal fibroblasts were pre-cultured for 3 days in vitro and then injected within a fibrin matrix into subcutaneous pockets on the dorsal flanks of SCID mice. Vessels deployed from these pre-cultured microbeads formed functional connections to host vasculature within 3 days and exhibited extensive, mature vessel coverage after 7 days in vivo. Cellular microbeads showed vascularization potential comparable to bulk cellular hydrogels in this pilot study. Furthermore, our findings highlight some potentially advantageous characteristics of pre-cultured microbeads, such as volume preservation and vascular network distribution, which may be beneficial for treating ischemic diseases.

## Introduction

Cardiovascular diseases (CVDs) are the most deadly and costly problems facing our healthcare system today, and their prevalence is increasing at an alarming rate. Atherosclerosis, characterized by the presence of fatty plaques, inflammation, and aberrant extracellular matrix (ECM) in arterial walls, is one form of CVD that is often asymptomatic and can lead to complete vessel occlusion and ischemia downstream of the lesions. Atherosclerosis in the coronary arteries is the leading cause of heart attacks, or myocardial infarction, but can also affect the peripheral vasculature with severe consequences. Approximately 18 million Americans suffer from peripheral arterial disease (PAD), and 2 million of these patients develop critical limb ischemia (CLI)—the end stage of lower limb PAD^1–4^. While invasive procedures (i.e., bypass surgery, stenting) can restore blood flow in atherosclerotic arteries, patients with co-morbidities may not be suitable candidates for such invasive surgeries. Hence, there is an urgent clinical need for new approaches to revascularize ischemic tissues without the need for open surgery.

Pro-angiogenic growth factors have been extensively studied in both pre-clinical models and clinical trials to promote the creation of new functional microvasculature and restore perfusion to ischemic tissues^5–7^. However, these therapies are limited in part by protein instability, the inability of single factors to faithfully recapitulate vascular development, and the inability to deliver multiple factors with precise spatiotemporal control^8^. As a consequence, clinical trials of therapeutic angiogenesis have been disappointing^9,10^. Cell-based approaches have also generated a great deal of enthusiasm, especially with the emergence of induced pluripotent stem cells and the ability to differentiate them into endothelial cells^11,12^. However, most cell-based approaches to-date have relied on delivery of cells alone, either intravenously or directly into a harsh ischemic microenvironment^13^, or embedded in a hydrogel biomaterial that gels in situ around the cells. In such cases, the delivered cells are expected to either promote host vessel invasion into the ischemic region or directly participate in vascular assembly.

An alternative strategy involves the creation of tissue constructs with vascular networks pre-formed in vitro that can be subsequently implanted into ischemic regions or used in the context of engineered tissues. Inosculation between host vessels and the vessels within pre-vascularized tissues has been demonstrated^14,15^, leading to improved viability and function of parenchymal cells after transplantation^16,17^. Similarly, in vivo pre-vascularization has also been achieved via the surgical implantation of avascular scaffolds close to an artery and/or vein, subsequent host vessel invasion within the implant over the time course of weeks, followed by surgically moving the now-vascularized tissue to the ischemic region where the implant and the host vessels are connected surgically via microsurgical anastomoses^18,19^. However, the invasiveness of possibly multiple surgical procedures makes such an approach undesirable and perhaps even infeasible for patients with CLI and other ischemic conditions.

Modular microtissues that can be delivered in a minimally invasive manner and subsequently self-assemble into macroscale vascularized networks in situ represent a new approach to recreate microvasculature in ischemic tissues that leverages the potential benefits of both pre-vascularized tissues and injectable delivery. The small sizes of modular microtissues (100–300 μm in diameter) ensure cells encapsulated within them are better sustained by nutrient and oxygen diffusion alone^20–24^. While a wide range of biomaterials have been fabricated into modular formats, here we fabricated tissue modules using fibrin, a naturally occurring biopolymer that promotes wound healing and neovascularization^25^ and is FDA cleared for some applications in humans^26^, such as a surgical sealant (e.g., Baxter’s TISSEEL and ARTISS). In prior work, we have shown that co-encapsulation of endothelial cells with stromal fibroblasts in modular microtissues (microbeads) fabricated from fibrin undergo a morphogenetic program akin to vasculogenesis inside the microbeads, and sprout outside the microbeads via angiogenesis when embedded in larger hydrogels that mimic surrounding tissue. Nascent endothelial sprouts also inosculate with sprouts from neighboring microbeads in 3D tissue models. Further, these microbeads have displayed high cell viability even after injection through a needle, demonstrating applicability for minimally invasive applications^27^. The purpose of this pilot study was to determine if microbeads can be pre-cultured for a period of time in vitro, subsequently delivered in vivo in a minimally invasive manner, and form functional connections with host microvasculature. Our findings demonstrate microbead fabrication and pre-culture do not have deleterious effects on vascularization potential in vivo. While vascularization potential of these microbeads was similar to that of cells uniformly distributed throughout bulk hydrogels, our findings demonstrate that pre-cultured microbeads delivered via injection are capable of forming functional microvasculature extensively distributed across tissue volumes. Our results also suggest cellular microbeads may have advantageous properties compared to cells delivered via bulk hydrogels, including implant volume preservation, enhanced vascular network distribution, and accelerated maturation, which may aid in improved revascularization of ischemic tissues.

## Results

### Microbeads support vascular morphogenesis

**Figure 1:**
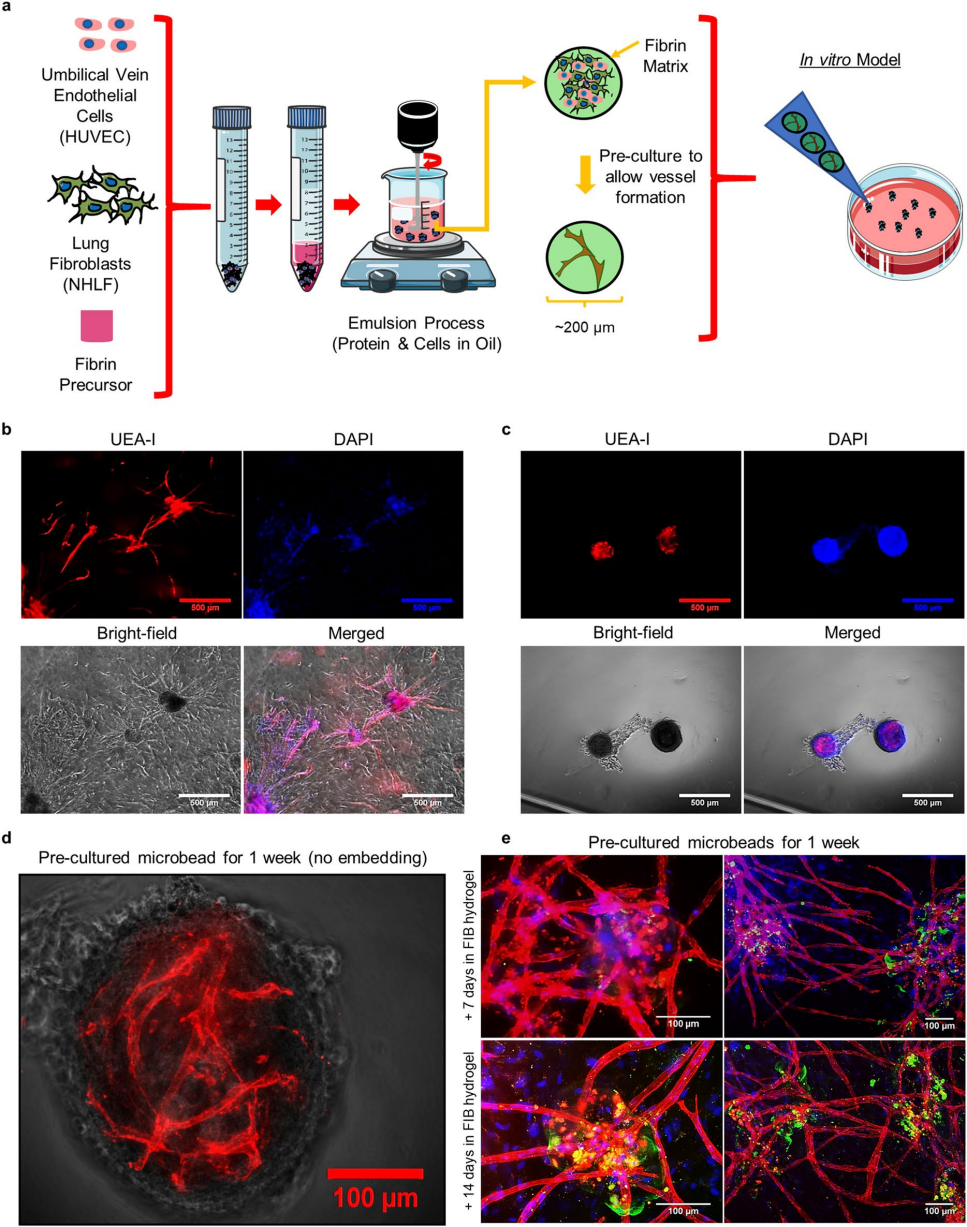
Fibrin microbeads support vascular morphogenesis. (**a**) Fibrin microbeads containing human umbilical vein endothelial cells (HUVEC) and normal human lung fibroblasts (NHLF) were made via an emulsification process. (**b**) Cell-containing microbeads embedded in fibrin gels immediately after fabrication catalyzed morphogenesis from the beads into the surrounding microenvironment at day 7. (**c**) Those cultured in suspension for 7 days supported both inter-bead and intra-bead morphogenesis. (**d**) A higher magnification image of a cell-laden microbead pre-cultured for 7 days in suspension shows intra-bead vascular morphogenesis. (**e**) Subsequent embedding of these pre-cultured microbeads in fibrin gels for an additional 7 (top row) or 14 days (bottom row) led to the formation of extensive interconnected inter-bead vascular networks initiated from the pre-cultured microbeads. Endothelial sprouts (red) were stained with UEA-I, nuclei (blue) were stained with DAPI, and microbeads (green) were labeled with FITC-fibrinogen (scale bar = 500 µm in (**b**) and (**c**), and 100 µm in (**d**) and (**e**)). Portions of this figure were created using images modified from Servier Medical Art (Servier, https://smart.servier.com, licensed under a Creative Commons Attribution 3.0 Unported License).

Fibrin microbeads containing a nominal 1:1 ratio of HUVEC and NHLF were made via an emulsification process, and either embedded within acellular fibrin hydrogels right away or after a period of pre-culture for up to 7 days (Fig. 1a). We selected a 1:1 ratio based on previous studies from our group and others^28–30^. HUVEC within the microbeads formed sprouts both within and outside the beads when embedded in acellular hydrogels immediately after fabrication, as previously reported^27^. Images from UEA-I stained cultures show HUVEC remained in the microbeads when cultured in suspension and sprouted from the beads to invade their surroundings once embedded within a larger fibrin hydrogel (Fig. 1b). Phase contrast images demonstrated that cells within microbeads pre-cultured in suspension for up to 7 days coalesced during the pre-culture period (Fig. 1c) and initiated cell–cell contacts in that context. Primitive microvascular networks formed within these microbead suspensions after 7 days (Fig. 1d). Subsequent embedding of these D7 microbeads in fibrin hydrogels (Fig. 1e) followed by an additional 7–14 days of culture yielded extensive vessel-like networks and functional connections between endothelial sprouts (red) coming from neighboring microbeads (FITC-labeled, green) as evidenced by observation of 3D image data sets and the presence of overlapping vessel-like structures in image projections. The additional 7 days of culture enabled maturation of the vessel-like networks and more extensive interconnectedness. Blue nuclei not contained within red HUVEC reveal the NHLF, demonstrating their presence throughout the model tissue (Fig. 1e). We have previously shown that NHLF adopt a mural cell-like localization around the vessel-like structures, and express a subset of pericyte markers^31–33^.

### Pre-culture time affects extent of vascular morphogenesis in vitro

**Figure 2:**
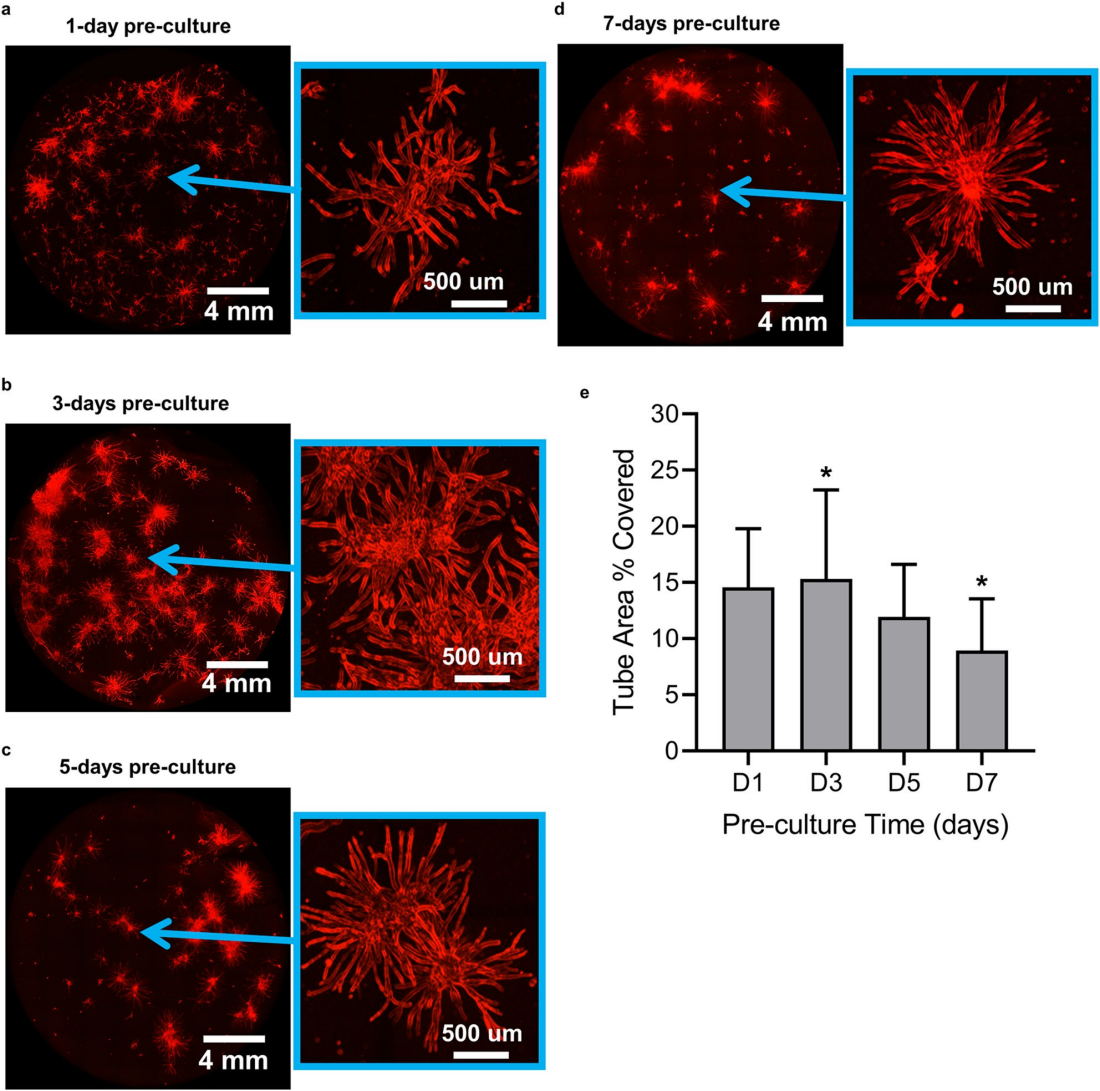
Pre-culture time affects vascular distribution in vitro*.* Microbeads were pre-cultured in suspension for (**a**) 1 day, (**b**) 3 days, (**c**) 5 days, or (**d**) 7 days, and subsequently embedded in fibrin hydrogels for an additional 7 days. Endothelial sprouts (red) were stained with UEA-I. Images on the right for each pair show regions of the hydrogel that were magnified and adjusted with a Kirsch filter to facilitate edge detection and quantification. (**e**) Quantification of the fractional area of each fibrin construct occupied by tubular structures as a metric of distribution showed that 3 days of pre-culture time led to the most extensive vascular networks. The symbol (*) on the graph indicates values were statistically different (*p* ≤ 0.05). Error bars indicate ± SD.

We next assessed if pre-culturing microbeads prior to embedding them caused any differences in the overall extent of the vessel-like networks formed in vitro. Microbeads containing HUVEC and NHLF were embedded in fibrin hydrogels after 1 (Fig. 2a), 3 (Fig. 2b), 5 (Fig. 2c), and 7 (Fig. 2d) days of suspension pre-culture, and subsequently cultured for an additional 7 days within the hydrogels. Each tissue construct was imaged in its entirety, and the fractional area occupied by endothelial tube-like structures was quantified. Extended periods of pre-culture beyond 3 days prior to embedding resulted in reduced coverage of the tubular structures within the fibrin hydrogel, indicative of a less extensive vessel-like network (Fig. 2e). Microbeads pre-cultured for 3 days produced significantly more extensive vascular networks compared to microbeads pre-cultured for 7 days (*p* < 0.05), and we therefore selected this pre-culture condition for subsequent in vivo studies.

### Cellular microbeads direct vascular morphogenesis in vivo

**Figure 3:**
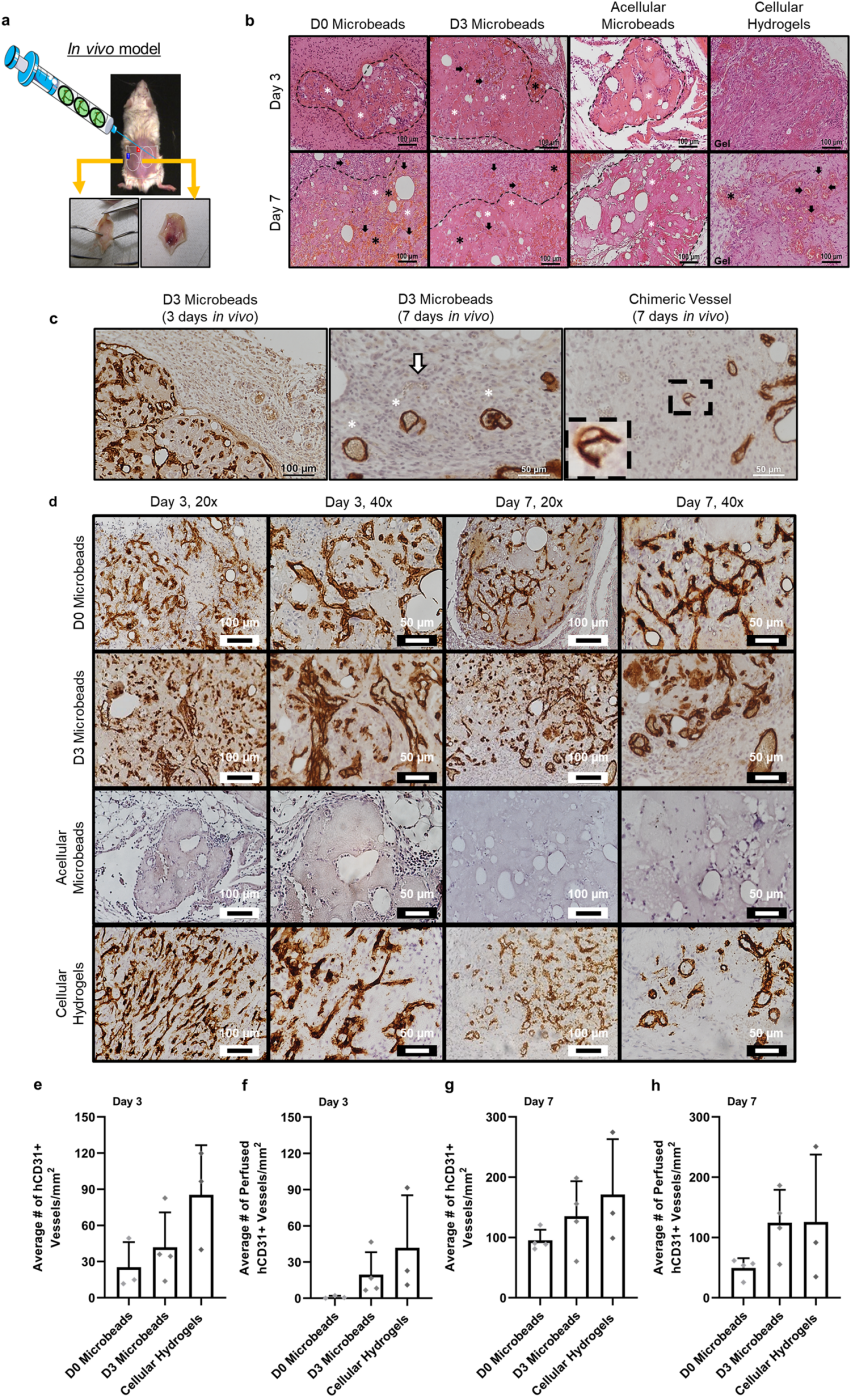
Cell-laden fibrin microbeads catalyze the formation of functional microvasculature in vivo. (**a**) Implants were injected into the subcutaneous space on the dorsal surface of SCID mice. (**b**) Implants were retrieved, fixed, processed, embedded, and then stained with hematoxylin and eosin. Representative images of H&E-stained sections show vessel formation and cell infiltration in D0 microbeads (first column), D3 microbeads (second column), acellular microbeads (third column), and cellular hydrogels (fourth column) for 3 days (top row) and 7 days (bottom row). Dashed lines highlight the clusters of microbeads located within the implant. Arrows indicate representative vessels, black asterisks indicate representative regions where host erythrocytes are clearly present, and white asterisks indicate representative individual microbeads. (**c**) Implants were also IHC-stained for hCD31 (dark brown) to confirm the human origin of the neovessels. Implants containing D3 microbeads evaluated in vivo for 3 (left image) and 7 (middle and right image) days showed evidence of inosculation with host vessels. The white arrow in the day 7 micrograph (middle image) identifies a perfused mouse vessel (hCD31-), while white asterisks highlight hCD31+ vessels with red blood cells. The dashed black rectangle suggests a chimeric vessel formed by both mouse and human cells (right image). The magnified inset demonstrates the presence of host erythrocytes within the vessel. (**d**) Vessel structures containing human endothelial cells (hCD31+) were identified in all implants except for those containing acellular microbeads. Shown are representative 20× and 40× images of implants stained for hCD31 after 3 and 7 days in vivo. Quantification of both total and perfused human EC-derived vessel density for each condition after (**e**,**f**) 3 days and (**g**,**h**) 7 days was performed. No significant differences were observed in vessel and perfused vessel density between any of the experimental groups containing human endothelial cells after 3 or 7 days in vivo. Individual data points on graphs represent a single implant quantified per condition. Error bars indicate ± SD. Portions of this figure were created using images modified from Servier Medical Art (Servier, https://smart.servier.com, licensed under a Creative Commons Attribution 3.0 Unported License).

Pre-cultured microbeads were then evaluated for their ability to form functional microvasculature in vivo. Microbeads containing HUVEC and NHLF pre-cultured in suspension for 3 days (D3 microbeads) were injected in an acellular fibrin precursor solution into subcutaneous pockets on the dorsal surface of SCID mice (Fig. 3a). Their ability to form functional microvasculature was compared to D0 microbeads (encapsulating HUVEC and NHLF without pre-culture), acellular microbeads, or cellular hydrogels (HUVEC and NHLF delivered in a fibrin precursor solution, which gels in situ). All implants containing human cells showed evidence of vascular morphogenesis after implantation for 3 and 7 days (Fig. 3b, Supplementary Fig. S1). HUVEC were identified in explanted tissue constructs via immunohistochemical (IHC) staining of human CD31 (Fig. 3c, brown). Host erythrocytes within the lumens of these hCD31+ neovessels confirmed the formation of functional anastomoses with host vasculature. Murine vessels (white arrows, negative for hCD31) were sometimes visible in sections counter-stained with hematoxylin. We also occasionally observed structures that simultaneously consisted of both human endothelial cells (in brown) and murine endothelial cells (not stained) in the same structure, suggestive of chimeric vessels (Fig. 3c, right panel, dashed rectangle). Representative images of hCD31+ sections of the four experimental groups at both days 3 and 7 and two different magnifications are shown (Fig. 3d). Quantification of the vessel and perfused vessel densities was achieved by counting the number of hCD31+ and erythrocyte-perfused hCD31+ structures with lumens, respectively. No significant differences in the average numbers of total hCD31+ vessels or perfused hCD31+ vessels per mm^2^ were observed across any of the implants containing cells at day 3 (Fig. 3e, f) and day 7 (Fig. 3g, h).

### Cellular microbeads form extensive functional vascular networks in vivo

**Figure 4:**
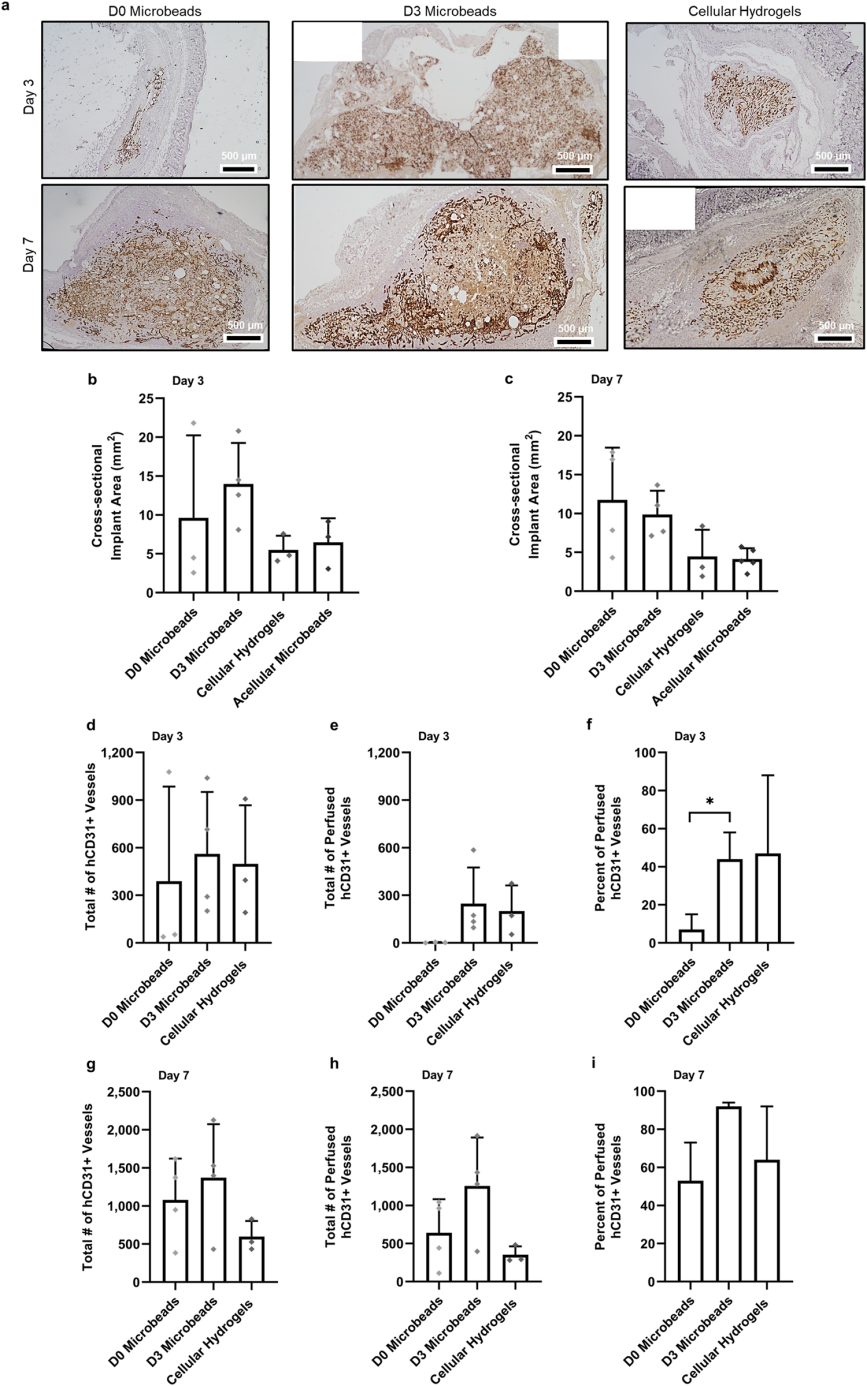
Cellular microbeads form extensive microvasculature in vivo. (**a**) Representative images of the cross-sectional areas of hCD31-stained tissues are shown for all 3 of the cell-containing experimental groups. D0 microbeads (left column), D3 microbeads (middle column), and cellular hydrogels (right column) were implanted for 3 (top row) and 7 days (bottom row) prior to their excision (black scale bar = 500 µm). Implant sizes were quantified by measuring the cross-sectional area of the implant region using H&E staining after (**b**) 3 and (**c**) 7 days in vivo. No significant differences in implant a rea were found between any of the experimental groups after 3 or 7 days. The total number of hCD31+ (**d**,**g**) vessels and (**e**,**h**) perfused vessel in the entire implant region was quantified after 3 and 7 days in vivo. No significant differences were observed between any of the cell-laden experimental groups after either time point. Percent of perfused hCD31+ vessels in the implant region after (**f**) 3 and (**i**) 7 days. After 3 days in vivo, D3 microbeads had a significantly greater percent of perfused vessel compared to D0 microbeads. After an additional 4 days in vivo, no significant differences were observed between any of the cellular conditions. Asterisks indicate statistically significant differences (*p* ≤ 0.05) between groups. Individual data points on graphs represent a single implant quantified per condition. Error bars indicate ± SD.

Implants containing cellular microbeads appeared larger in volume upon extraction from the mice. To evaluate this observation, implants were histologically sectioned through their entire volume, each section (6 µm) was stained for H&E, and then the section with the maximum area (approximating the center of the implant region) was identified by quantifying stitched images of the entire implant region (Fig. 4a). Using this approach, the implant area (in mm^2^) and the total number of vessels in the maximum implant area were quantified in tissues explanted after 3 and 7 days in vivo (Fig. 4b–i). While trends in the data suggest implants containing cellular microbeads had larger average cross-sectional areas than both acellular microbeads and cellular hydrogels on day 3 and 7, these differences were not statistically significant (Fig. 4b, c). With the exception of D0 microbeads, the average implant cross-sectional area of all conditions decreased from day 3 to day 7 in vivo.

We then multiplied these implant areas by the average vessel density to calculate the total numbers of vessels within the implant groups, and found all cellular implants contained comparable total numbers of hCD31+ vessels after 3 days in vivo (Fig. 4d, e). D0 microbeads still showed minimal evidence of vessel perfusion on day 3 even when considering total vessel coverage throughout the entire implant (Fig. 4e, f). For all conditions, the total numbers of hCD31+ vessels and hCD31+ perfused vessels increased from day 3 to day 7 (Fig. 4g, h); however, this increase was notably smaller in cellular hydrogels, which only showed a 1.2-fold increase in total number of vessels from day 3 to day 7 in vivo compared to a 3.31-fold and 2.44-fold increase in D0 and D3 microbeads, respectively. Implants containing D0 and D3 microbeads also exhibited greater increases in the total numbers of perfused vessels (233.9-fold and 5.1-fold, respectively) from day 3 to day 7 than cellular hydrogels (only 1.8-fold). Furthermore, of the total number of hCD31+ vessels formed by day 7 (Fig. 4i), 91.8% of vessels in D3 microbead implants were perfused, compared to only 54.8% and 64% in D0 microbeads and cellular hydrogels, respectively.

### Cellular implants attain α-SMA expression in vivo

**Figure 5:**
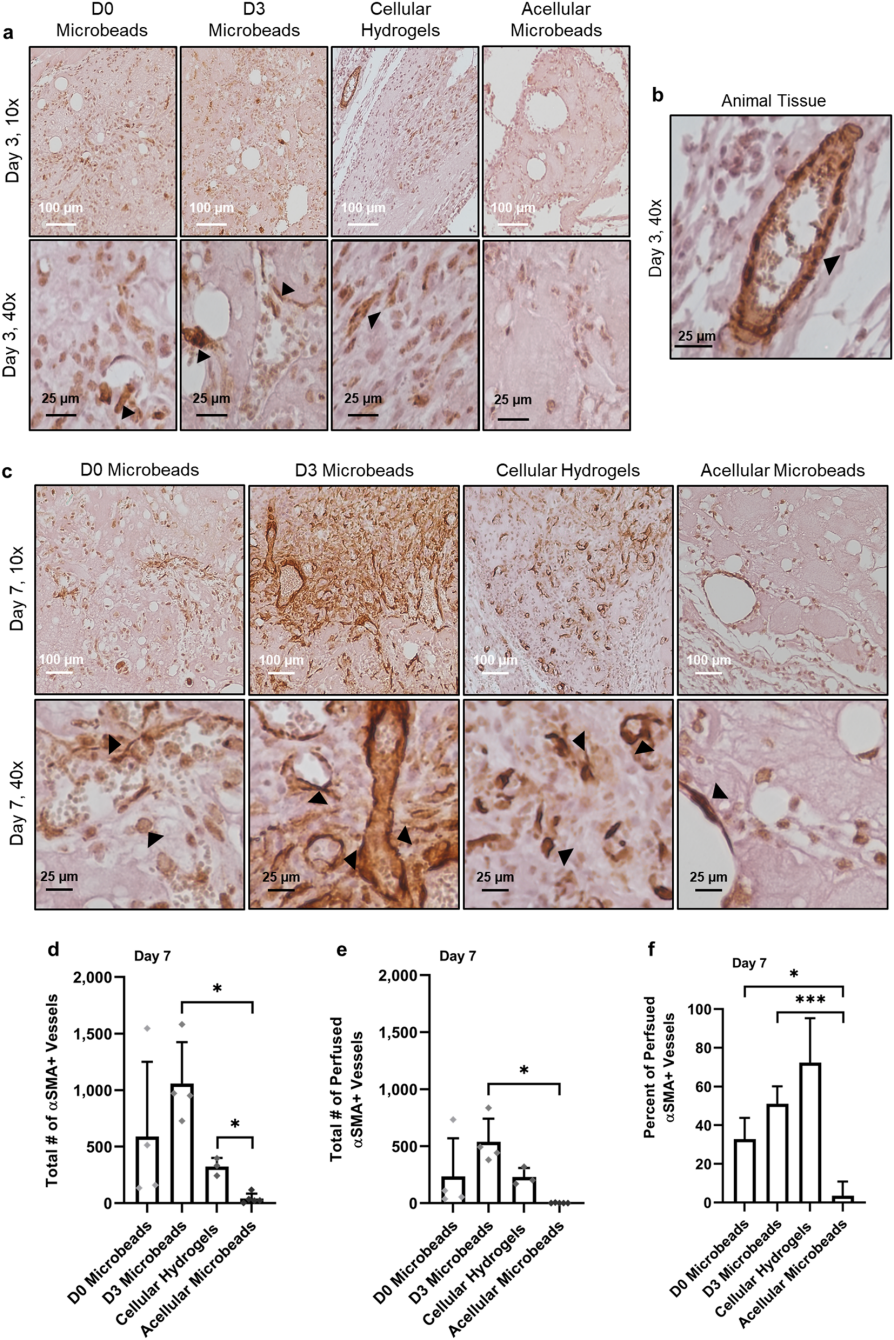
Implants containing cell-laden microbeads contained α-SMA supported vessels. (**a**) D0 microbeads (first column), D3 microbeads (second column), cellular hydrogels (third column), and acellular microbeads (fourth column) that were injected and kept in vivo for 3 days displayed minimal evidence of α-SMA+ staining. Images acquired via 10× (top row) and 40× (bottom row) objectives highlight α-SMA staining in brown (black arrows). (**b**) Vessels within the surrounding host tissue were positive for α-SMA as shown by 40× image from animal tissue near 3-day cellular hydrogel implant. (**c**) After 7 days, expression of α-SMA positive structures was higher in all conditions. White scale bar = 100 µm, and black scale bar = 25 µm. Quantification of total number of (**d**) α-SMA+ and (**e**) perfused α-SMA+ vessels in the implant region after 7 days in vivo. D3 microbeads had significantly higher α-SMA+ vessel and perfused vessel densities, and total number of perfused α-SMA+ vessels than acellular microbeads. D3 microbeads and cellular hydrogels both had a higher total number of α-SMA+ vessels than acellular microbeads. (**f**) Percent of perfused α-SMA+ vessels in the implant region after 7 days. Both D0 and D3 microbeads had a significantly greater percent of perfused vessel compared to acellular microbeads. Asterisks indicate statistically significant differences (*p* ≤ 0.05) between groups. Individual data points on graphs represent a single implant quantified per condition. Error bars indicate ± SD.

IHC staining of smooth muscle alpha-actin (α-SMA), a pericyte marker, was also performed to determine the extent of vessel maturation across the different experimental groups. At the day 3 time point, α-SMA expression (brown stain with black arrows) was sparse in all four experimental groups, even when images were magnified to better visualize stained structures (Fig. 5a). Most positive α-SMA staining observed within the implant region at this early time point was diffuse and not necessarily localized subjacent to vessel structures. Positive α-SMA staining around vasculature was readily observed in the host tissue localized outside of the implant region, displayed at the top left corner of the cellular hydrogel image in Fig. 5a (magnified in Fig. 5b). After an additional 4 days in vivo, cellular conditions all displayed stronger α-SMA staining compared to the day 3 time point (Fig. 5c). Implants containing acellular microbeads exhibited minimal α-SMA+ staining within the implant region (Fig. 5c). Quantification of vessels stained positive for α-SMA showed the extent of vessel maturation differed significantly only between cellular conditions and acellular microbeads (Fig. 5d–f). Specifically, D3 microbead and cellular hydrogel groups both had a significantly higher total numbers of α-SMA+ vessels than acellular microbeads (*p* = 0.04 for both conditions) (Fig. 5d). Quantification of the total number of perfused α-SMA+ vessels revealed the D3 microbead group had the highest numbers of these mature vessels, although the only significant difference was observed compared to the acellular microbead group (*p* = 0.05) (Fig. 5e). D0 and D3 microbead conditions also had a significantly higher percent of α-SMA+ vessels perfused compared to acellular microbeads after 7 days in vivo (Fig. 5f).

### Volume preservation of cellular microbeads may influence maximum implant area and vascular network distribution

**Figure 6:**
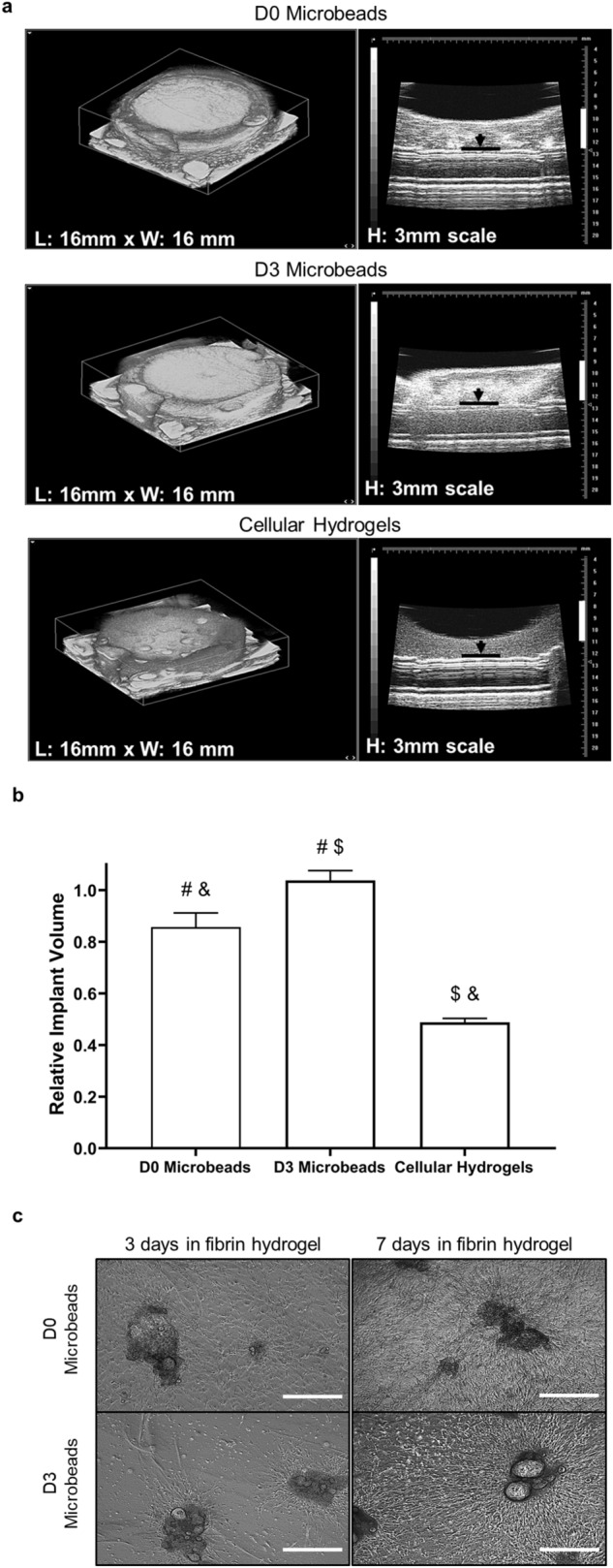
Constructs with D3 microbeads do not compact after 1 day of in vitro culture. (**a**) 3D ultrasound images of model fibrin implants containing D0 microbeads (top row), D3 microbeads (middle row), or uniformly suspended cells (cellular hydrogels, bottom row). Black lines and arrows in the images on the right-hand side show the bottom of the implant; white vertical lines are 3 mm scale bars. (**b**) Relative implant volumes were quantified and normalized to the model implants containing D3 microbeads, whose volumes were constant after 24 h of in vitro culture. Statistically significant differences (*p* ≤ 0.05) are indicated by matched symbols. Constructs containing D3 microbeads were of significantly larger volume than those containing D0 microbeads or the cellular hydrogels. (**c**) Cells deployed more slowly from D3 microbeads when embedded in fibrin hydrogels. Bright-field images of D0 (top row) and D3 (bottom row) microbeads embedded in fibrin hydrogels for 3 (left column) and 7 days (right column). Scale bar = 500 µm. Error bars indicate ± SD.

The observed differences in implant morphologies suggested the cellular microbead formulations may yield larger implants in part because of their ability to better preserve implant volume over time. While the cellular hydrogel group exhibited higher hCD31+ vessel densities, the total numbers of hCD31+ vessels across the implants trended highest in the cellular microbead groups after 7 days in vivo, suggesting microbead formulations may be particularly useful in creating a more distributed vascular network throughout the entirety of an implant. To assess this directly, implant volumes were measured in model constructs in vitro the day after fabrication via 3D high-resolution ultrasound imaging (Fig. 6a). Fibrin-based constructs containing D3 microbeads did not significantly change volume within 24 h. By contrast, the volumes of constructs containing D0 microbeads or uniformly distributed cells (“cellular hydrogels”) significantly compacted, attaining volumes that were 17% and 47% less, respectively, than those containing D3 microbeads after only 24 h of incubation in vitro. All measured volumes were statistically different from one another (*p* ≤ 0.05, Fig. 6b). Further, we investigated if differences observed between microbead conditions could be attributed to cell invasion from microbeads into the surround fibrin hydrogel, leading to matrix remodeling and implant compaction. Phase contrast images qualitatively showed fewer cells migrated out of D3 microbeads than D0 microbeads in the surrounding fibrin matrix, especially at early time points (Fig. 6c). This observation is clearer when microbeads are left in the fibrin hydrogels for 3 days rather than 7 days since there is less time for cells to remodel the matrix. Collectively, these data reveal that constructs containing cellular microbeads exhibited less compaction, potentially due to delayed cell invasion and matrix remodeling, relative to cellular hydrogels, which may influence maximum implant area and vascular network distribution observed in vivo.

## Discussion

Creating functional microvasculature remains a major challenge to engineering functional tissue replacements and restoring function in many ischemic pathologies. In this proof-of-concept study, we examined an approach to engineer microvasculature in vivo based on sub-millimeter vascularized tissue modules (microbeads) that can be injected via syringe in a minimally invasive manner. Similar approaches based on cell spheroids^34–42^, microgels containing cells^43–45^, and modular microtissues with an exterior coating of endothelial cells^23^ have all shown microvascular potential in vitro and in vivo. Inspired by these and other previous studies, our approach here preserves many of the attractive features of macroscale pre-vascularized tissue constructs^14–17^, including the flexibility to be fabricated with a range of materials and cell types^27,32,46^ and the ability to support the complex morphogenetic programs necessary to recreate microvasculature that can inosculate with host vasculature following delivery in situ*.* While other studies have investigated the use of scaffold-free multicellular aggregates for vascularization applications^34–42^, the use of ECM proteins for cell encapsulation provides an immediate 3D matrix conducive to remodeling during vascular assembly, which may be beneficial for more rapidly producing robust vascular networks throughout microbeads prior to implantation in vivo. Fibrin was chosen as the material for our microbeads for applications in tissue vascularization because it improves cell survival during injection^25^, promotes wound healing and endothelial morphogenesis^27,47^, and is cleared by the FDA for use as a surgical sealant in humans^26^. Additionally, fibrin’s microstructure and polymerization rate can be readily modified^26^.

Consistent with our prior study^27^, we first demonstrated here that HUVEC co-encapsulated with NHLF in fibrin microbeads were capable of sprouting into a surrounding fibrin matrix and forming interconnected networks of vessel-like structures. We then assessed the value added by pre-culture prior to either embedding the microbeads in a model tissue in vitro or injecting them in vivo. Cells encapsulated in microbeads held in static suspension culture without embedding in an additional matrix for up to 7 days were able to form primitive vessel-like structures within each bead. Once embedded, endothelial sprouts from adjacent pre-cultured microbeads appeared to form connections with each other within the surrounding fibrin matrix. The distribution of the microvasculature nucleated by the microbeads embedded in larger model tissues (quantified by the area fraction occupied by endothelial tubules) was significantly improved when microbeads were pre-cultured for 3 days prior to embedding. Longer pre-culture times led to less extensive and more heterogeneous networks, perhaps due to microbead aggregation during pre-culture, which may have resulted in less uniform distribution of microbeads in the matrix. The observed formation of vessel-like structures within and between microbeads is consistent with other studies using endothelial and stromal supportive cells encapsulated in microgels of varying matrices^43–45^. Other studies have found longer pre-culture times (up to 14 days) beneficial, especially when compared to non-pre-cultured controls^43,44^; however, optimal pre-culture time is influenced by cell type, cell density, and material properties of the microgels.

The in vivo vascularization potential of these microbeads pre-cultured for 3 days (D3 microbeads) was then compared to D0 microbeads (containing HUVEC and NHLF but no period of pre-culture), acellular microbeads, and cellular hydrogels (HUVEC and NHLF delivered in a fibrin precursor solution, which gels in situ). All four experimental groups were delivered as injectable formulations, with microbeads being delivered within an acellular fibrin precursor solution, into subcutaneous pockets on the dorsal surface of SCID mice. Pre-culturing microbeads for 3 days prior to delivery did not significantly accelerate the formation of anastomoses with host vessels in this particular animal model, as all three groups containing cells showed evidence of hCD31+ vessel formation by day 3. Further, at this time point D3 microbeads and cellular hydrogels also showed evidence of functional perfusion (i.e., the presence of erythrocytes). D0 microbeads showed little evidence of perfusion by day 3, perhaps indicating that the fabrication process of the microbeads may result in the encapsulated cells needing additional time to equilibrate to the new matrix prior to vessel morphogenesis, causing a delay in their ability to anastomose with host vasculature. After 7 days in vivo, all cellular implants contained perfused hCD31+ vessels. All three cellular conditions showed an increase in total number of vessels and perfused vessels from day 3 to day 7 in vivo, however, increases observed in cellular hydrogels were much lower than those of cellular microbeads. Though cellular hydrogels had greater average hCD31+ vessel densities, the total number of hCD31+ vessels throughout the entire implant region trended higher in D0 and D3 microbead conditions after 7 days, likely due to the larger sizes of cellular microbead implants. The total number of perfused hCD31+ vessels also trended higher for the D3 microbead group than D0 microbeads and cellular hydrogels. Interestingly, in a similar study utilizing pre-cultured multicellular alginate microgels implanted in a fibrin plug, non-pre-cultured (D1) microgels failed to assemble into vascular networks in the subcutaneous space after 7 days. Pre-cultured (D14) microgels showed some evidence of vessel perfusion after 7 days, but required an additional 7 days in vivo to show significant evidence of vessel perfusion throughout the implant^44^.

Further, tissue sections were also stained for α-SMA to characterize pericyte coverage and vessel maturity within the implants. Mature vessels were only found in the surrounding mouse tissue on day 3, but by day 7 all cellular implants exhibited higher levels of α-SMA expression. Acellular microbeads had minimal α-SMA expression throughout the implant even at the day 7 time point indicating minimal infiltration of host vasculature. All cellular conditions had comparable α-SMA+ vessel densities and total number of α-SMA+ vessels after 7 days in vivo, but only D3 microbeads had a significantly higher average α-SMA+ vessel and perfused vessel density compared to acellular microbeads. D3 microbeads were also the only group to have a significantly higher total number of perfused α-SMA+ vessels compared to acellular microbeads. This could be a result of pro-angiogenic growth factor sequestration to the fibrin matrix of microbeads during pre-culture. These growth factors could aid in the recruitment of mature host vessels into the implant region upon delivery of pre-cultured microbeads in vivo, leading to greater expression of α-SMA throughout the implant. Further experiments are needed to more definitively determine if the α-SMA+ mural cells observed around the vessels were due to host vasculature infiltration or differentiation of the NHLF into bona fide pericytes, although previous evidence from our group suggests NHLF only express a subset of pericyte markers, at least in our in vitro models^31^.

The larger size of implants containing cellular microbeads translated to trends of higher numbers of total vessels across the entire implant volume, likely resulting in a more extensive vascular distribution. As observed differences in implant area were not statistically significantly, possibly due to the inherent variability in sample collection and histological processing, we used ultrasound to quantify the volumes of model implants cultured in vitro. A phenomenon similar to the compaction observed in vivo was also observed in these in vitro constructs. Constructs containing D3 microbeads did not compact, while those with D0 microbeads compacted approximately 20%. By contrast, the cellular hydrogel constructs, in which the cells were initially dispersed uniformly, compacted ~ 50% in just 24 h. Furthermore, constructs containing D3 microbeads contained higher density features (presumably the cell-laden beads) based on the higher attenuation displayed in the ultrasound images relative to the other constructs. Cells encapsulated in the D3 microbeads also took longer to infiltrate the surrounding fibrin hydrogel than the ones residing in the D0 microbeads, perhaps due to increased local ECM density^48^. The volume preservation of constructs containing cellular microbeads, combined with the delayed deployment of cells from these beads, may underscore the utility of cellular microbead formulations to create more distributed microvascular networks.

In summary, this study demonstrated that fibrin microbeads containing cellular building blocks of microvasculature readily vascularize a fibrin construct in subcutaneous tissue and support a high degree of vascularity comparable to cellular hydrogels. This finding demonstrates that the fabrication and subsequent pre-culture of cellular microbeads do not have deleterious effects on the ability of these microbeads to form functional vasculature upon implantation in vivo. While other studies have investigated the use of cells spheroids and microgels for vascularization applications, few have utilized ECM protein-based microbeads for the encapsulation of multiple cell types to create discrete units for vascularization. Though differences observed between experimental groups were often not significant, the data suggest that cellular microbeads may have advantageous properties over cells uniformly distributed throughout bulk hydrogels, such as implant volume preservation and enhanced vascular network distribution. Pre-culturing microbeads may accelerate vessel inosculation with host vasculature and maturation characterized by an increase in the total numbers of pericyte-invested perfused microvessels. However, while the subcutaneous model is straightforward and commonly used to evaluate vascularization strategies in vivo, it does not mimic the harsh ischemic conditions typical of many clinical conditions. This proof-of-concept study therefore lays the groundwork for higher-powered animal studies in more physiologically-relevant models of ischemia, where cell survival may be more challenging due to the lack of oxygen and nutrients. Whereas encapsulated cells might not survive long enough to form vessels de novo, primitive microvasculature deployed from injectable pre-cultured microbeads may better withstand harsh ischemic environments and nucleate a distributed vascular network that can rapidly inosculate with host vessels to support parenchymal cells for regenerative applications.

## Materials and methods

### Cell culture

Human umbilical vein endothelial cells (HUVEC) were either purchased from a commercial source (Lonza, Inc., Walkersville, MD) or isolated from umbilical cords from the University of Michigan Mott Children’s Hospital as previously described^47^. [Umbilical cords were obtained by a process considered exempt by the University of Michigan’s Institutional Review Board (notice of determination dated August 21, 2014) because the tissue is normally discarded, and no identifying information is provided to the researchers who receive the cords.] These two different HUVEC sources were evaluated in vitro to demonstrate the robustness of the system, regardless of endothelial source, as shown in the past^46^. HUVEC isolated from fresh cords were used for in vitro studies and the commercial HUVEC were used for the in vivo studies. This was due to limited availability of fresh cords for HUVEC isolation. Purchasing HUVEC from a commercial source ensured all experimental condition contained HUVEC from a single source to prevent variability in in vivo data due to cell sourcing. Regardless of source, HUVEC were grown in endothelial growth media (EGM-2, Lonza), and used from passages 4–7. Normal human lung fibroblasts (NHLF) were also purchased (Lonza), cultured in Media 199 (M199, Life Technologies, Grand Island, NY) with 10% fetal bovine serum (FBS), and used from passages 9–14 for in vitro and in vivo experiments.

### Fibrin microbead production

Fibrin microbeads were made in a water-in-oil emulsification process, as previously described^27^. Prior to starting the emulsification process, 75 mL of 100 cSt polydimethylsiloxane (PDMS) oil (Clearco Products Co. Inc., Bensalem, PA) was placed into a 100 mL beaker and left on ice. Cells were pelleted in a 1:1 HUVEC:NHLF ratio in a conical tube at the initial cell density of 2 × 10^6^ total cells/mL (in vitro studies) or 5 × 10^6^ total cells/mL (in vivo studies). Lower cell concentrations were used for the in vitro studies to facilitate quantification of vessel-like structures. Each batch of microbeads (3 mL total volume) was made by mixing 765 µL of serum-free endothelial growth media (SFEGM-2), 300 µL of fetal bovine serum (FBS, 10% final), 60 µL of 50 U/mL thrombin (1 U/mL final), and 1875 µL of fibrinogen stock solution (2.5 mg/ml final clottable protein) with the HUVEC-NHLF pellet. The cell and protein mixture was then added to the PDMS bath, which was kept on ice at 0 °C. Bovine fibrinogen (FGN, Sigma Aldrich, St. Louis, MO) stock solution was made by mixing protein with SFEGM-2 at 37 °C. The cell-fibrin solution was mixed at 600 RPM for 5 min at 0 °C to allow for emulsion, and then mixed for 25 min at 37 °C to allow for gelation. The microbeads and PDMS solution were collected and separated with the addition of phosphate buffer saline (PBS) with 100 ppm of L101 surfactant (BASF, Florham Park, NJ) for inversion mixing, followed by 4–5 centrifugation steps (200 g for 5 min/each). After each centrifugation step, PDMS was removed from the fibrin microbeads. The microbeads were then re-suspended with EGM-2 and placed in vented 15 mL conical tubes with filters (CELLTREAT Scientific Products, Shirley, MA) prior to starting any experimental procedures. Medium was changed the day after microbead fabrication then every other day after.

### Embedding of microbeads in fibrin hydrogels

Fibrin microbeads were embedded in fibrin hydrogels immediately, 1, 3, 5, or 7 days after their fabrication process. One tenth of a 3 mL microbead stock was utilized to make 3 fibrin hydrogels, with ∼ 100 μL of microbeads per hydrogel. Constructs were made by mixing the microbead pellet thoroughly with 382.5 μL of SFEGM-2, 150 μL of FBS (10% final), 30 μL of 50 U/mL thrombin (1 U/mL final), and 937.5 μL of fibrinogen stock solution (2.5 mg/mL final clottable protein). 500 μL of the microbead-protein mixture was added per well of a standard 24-well culture plate, and left at room temperature for 5 min before being placed in the incubator for 25 min at 37 °C. EGM-2 (1 mL/well) was added to each hydrogel after the complete gelation process. Medium was replaced the next day then every other day after for the duration of the culture. In some experiments, microbeads were labeled with FITC-fibrinogen prior to embedding as previously shown^27^.

### Endothelial cell staining, imaging, and quantification

Prior to staining, microbeads alone or embedded in fibrin hydrogels were fixed in zinc-buffered formalin (Z-Fix, Anatech, Battle Creek, MI). After 10 min, the fixative was removed and the samples were washed 2× with PBS. The endothelial cell specific lectin Ulex Europaeus Agglutinin I (UEA-I, Vector Laboratories, Burlingame, CA) was utilized to stain the HUVEC. A staining solution consisting of 1% BSA, 20 µg/mL rhodamine-labeled UEA-I, and 10 nM DAPI in PBS was incubated with microbead samples and left for 45 min at room temperature. Samples were then washed with PBS 2–4× prior to imaging. Hydrogels were taken out of the wells of a 24-well plate, placed on a microscope slide (Superfrost Plus Microscope Slides, Fisher Scientific, Pittsburgh, PA), and then overlaid with a microscope cover glass (Vista Vision Cover Glass, VWR International, LLC, Radnor, PA). The scan slide tool in the Olympus software (IX2-BSW, version 01.07, Olympus, Center Valley, PA) and an optical microscope (Olympus IX81, Olympus) was used to take single plain fluorescent images of endothelial networks formed by fibrin microbeads embedded in fibrin hydrogels. To evaluate microbead characteristics, both after continuous suspension culture and culture in a larger fibrin hydrogel, z-stacks of areas of interest were acquired and flattened to produce maximum intensity projection images. ImageJ (National Institutes of Health, Bethesda, MD) was utilized to merge fluorescent and bright-field images. Prior to quantification, all endothelial images were cropped and processed using the Kirsch filter to detect edges of endothelial sprouts. The processing and imaging settings were kept constant for all conditions. The Angiogenesis Tube Formation module in Metamorph Premier imaging software (https://www.moleculardevices.com/products/cellular-imaging-systems/acquisition-and-analysis-software/metamorph-microscopy, version 7.8.6.0, Molecular Devices, Sunnyvale, CA) was then used to quantify the area of each fibrin hydrogel occupied by endothelial tubules as a metric of microvascular network coverage. At least 12 individual hydrogels per pre-culture condition were quantified to assess differences between experimental groups.

### Sample preparation for subcutaneous injection

Subcutaneous implants consisted of 2 × 10^6^ cells (HUVEC:NHLF in a nominal 1:1 ratio) in 500 µL of a fibrin precursor solutions for all 3 experimental groups containing cells. To ensure the same numbers of cells were injected into each mouse across different microbead preparations, a method was developed to quantify the number of cells within a given volume of microbeads after fabrication (as opposed to relying only on the theoretical starting cell concentration, as is more typical), and the volume of cell-laden beads injected into each mouse was adjusted in order to ensure consistent delivery of 2 × 10^6^ cells per implant. Briefly, microbead pellets from each batch were re-suspended in 1 mL of EGM-2 and transferred into vented 15 mL conical tubes with filters (CELLTREAT) and either processed immediately or cultured for 1 day. The total volume of microbeads and media was noted, and then 100 µL was transferred to a 96-well plate. An equal volume (100 µL) of a solution containing the fibrinolytic enzyme nattokinase (NSK-SD, Japan Bio Science Laboratory Co., Ltd) at a concentration of 50 FU/mL (fibrin degradation units) in D-PBS (calcium- and magnesium-free) containing 1 mM EDTA^49^ was then added on top and mixed. The plate was incubated at 37 °C for 30 min to allow the microbeads to degrade. Following fibrinolysis, cells were counted using a hemocytometer, and the microbead volume preparations adjusted to yield 2.4 × 10^6^ total cells, assuming half HUVEC and half NHLF. Samples for all 4 experimental groups (control microbeads, pre-cultured microbeads, acellular microbeads, or cellular hydrogels) were then centrifuged at 200 g for 1–5 min to form cell or microbead pellets, supernatant removed, and the pellets resuspended in 600 µL of fibrin hydrogel precursor solution comprising 270 µL of SFEGM-2, 60 µL of FBS (10% final), 12 µL of thrombin (1 U/mL final), and 258 µL of fibrinogen stock solution (2.5 mg/mL final clottable protein). Each solution was rapidly mixed, transferred into a 1-mL syringe fitted with a BD PrecisionGlide 20G needle, and 500 µL of the 600 µL solution was subsequently injected into the animal to deliver 2 × 10^6^ total cells per implant.

### Subcutaneous injections

All animal procedures were compliant with the NIH Guide for Care and Use of Laboratory Animals and approved by the University of Michigan’s Institutional Animal Care and Use Committee (IACUC). Male C.B-17 SCID mice, 6–8 weeks of age, (Taconic Labs, Hudson, NY) were acclimated for ≥ 72 h prior to surgery. An anesthetic/analgesic drug mixture of ketamine (80–120 mg/kg), xylazine (5–10 mg/kg), and buprenorphine (0.05–0.01 mg/kg) was administered to each animal via intraperitoneal injection. Ophthalmic ointment (Puralube Vet Ointment, Dechra, Overland Park, KS) was added to the eyes of each mouse. Dorsal lumbar flanks were shaved and depilatory agent (Nair, Fisher Scientific, Pittsburg, PA) was applied to remove remaining hair. The injection sites were sterilized by wiping the flanks 3× with alternating ethanol and Betadine (Thermo Fisher Scientific, Fremont, CA). A sterile surgical field was created to place the syringes, needles, and forceps on prior to injection. The samples were prepared, mixed, and injected subcutaneously on the dorsal flanks of the mouse with sterile gloves. The needle was left in the injection site for 30 s to allow for initial gelation of the solution prior to removal. Mice were allowed to recover from the anesthesia before being placed in their normal housing. The following four experimental groups were evaluated: (1) Acellular microbeads, (2) cellular hydrogels, (3) D0 microbeads (no pre-culture), and (4) D3 microbeads (pre-cultured for 3 days post-fabrication). Microbeads in all conditions were delivered in 500 µL of acellular fibrin precursor solution which gelled in situ. Bilateral implants were injected per animal, one on each flank in a randomized fashion. A total of 20 mice (n = 5 implants/condition, 2 implants/mouse) were used for this study. A second dose of buprenorphine (0.05–0.01 mg/kg) was administered to each animal 12 h after subcutaneous injections. Animals were monitored every day post-surgery.

### Implant retrieval and post-processing

After 3 or 7 days, animals were euthanized. Implants were located, removed with scissors and forceps, placed immediately in 20 mL glass scintillation vials with Z-fix, and subsequently fixed for 24 h at 4 °C. In some cases, implants could not be reliably located in the subcutaneous space after 3 or 7 days, presumably due to rapid fibrinolysis, and therefore not all formulations resulted in implants that could be retrieved for sample processing. Implants that could not be reliably retrieved were not included in analyses. After fixation, implants were washed with PBS 3–4× (5 min/wash), submerged in 70% ethanol, and stored at 4 °C until further processing. Samples were then placed in pink cassettes (Unisette Tissue Cassettes, Simport, Canada), embedded in paraffin in a KD-BMII tissue embedding center (IHC World, Ellicott City, MD), and sectioned through their entire volume with a Thermo Scientific HM 325 rotary microtome (6 µm sections) for further analysis.

### Hematoxylin and eosin (H&E) staining

Sections were stained with Mayer’s hematoxylin (Electron Microscopy Sciences, Hatfield, PA) and eosin Y (Sigma). Slides were dewaxed with xylene twice (5 min/wash), and then transferred to 100%, 95%, 70% ethanol, and deionized water baths (3 min/wash, two baths per ethanol concentration). Slides were submerged in hematoxylin bath for 15 min, and then rinsed with tap water for an additional 15 min. Slides were then placed in 95% ethanol for 30 s, followed by their immersion in eosin for 1 min. Slides were subsequently transferred into 95% ethanol bath for 1 min, and 2 separate 100% ethanol baths (1 min/bath). Samples were cleared by submerging them into 2 xylene baths (3 min/wash). Toluene mounting solution was added to each slide prior to placing the coverslips. Slides were left to dry overnight prior to imaging.

### Human CD31 (hCD31) and alpha-smooth muscle actin (α-SMA) staining

The largest cross-sectional area of each implant (approximating the center of the implant region) within the explanted tissue sections was first identified with H&E staining. The subsequent serial section of each implant was then deparaffinized with xylene and rehydrated through a series of graded ethanol washes and ending with water. Slides were placed in antigen retrieval solution (Dako: Agilent, Santa Clara, CA) and placed in steamer (95–99 °C) for 35 min. The antigen retrieval solution with the samples was then removed and slides equilibrated to room temperature. Slides were rinsed 3× with tris-buffered saline (TBS-T), 2 min/wash. The area around the tissue was marked with an ImmEdge pen (Vector Laboratories, Inc., Burlingame, CA). The Dako EnVision System-HRP (DAB) kit (Dako) was utilized for hCD31 and α-SMA staining. Slides were rinsed with TBS-T (3 times, 2 min/wash) before any solution from the Dako kit was added to the samples. First, peroxidase blocking solution was added to each tissue for 5 min. For hCD31 labeling, a mouse anti-human CD31 monclonal antibody (Dako, 1:50 dilution in TBS-T) was used as the primary antibody, incubated at 4 °C for 16 h. For α-SMA labeling, a mouse anti-alpha smooth muscle actin monoclonal antibody (1A4 (asm-1)) (Invitrogen: Thermo Fisher Scientific) was used as the primary antibody, incubated at room temperature for 2 h. The HRP-labeled polymer solution was added to each sample and left for 30 min. Samples were then kept with DAB+ substrate-chromogen buffer solution for 5 min. At least one sample from each experimental condition was subjected to the entire staining protocol without primary antibody as a negative control to confirm the specificity of the staining. Most samples were counter-stained with hematoxylin and/or eosin. Slides were then washed with 95% ethanol, 100% ethanol, and xylene. Toluene mounting medium was added prior to covering the samples with coverslips.

### Vessel quantification

Bright-field images (4× and 20×) of each complete implant stained for H&E, hCD31, and α-SMA were taken with an inverted Nikon microscope (Nikon Instruments Inc., Melville, NJ). Auto stitch software, developed by Brown and Lowe^50^, was utilized to create complete images of each implant from the 4× images taken. The total cross-sectional area (mm^2^) of each implant was quantified using ImageJ after first defining the perimeter of the implant using H&E staining. For hCD31+ vessel quantification, five 20× hCD31-stained images from each implant were quantified to determine the average vessel density (# of vessels/mm^2^). The cross-sectional area (mm^2^) was then multiplied by the average vascular density (# of vessels/mm^2^) to determine the total number of hCD31+ vessels found within each implant at days 3 and 7. For α-SMA+ vessel quantification, 20× images were taken of the entire implant and all images were quantified to determine the total number of vessels in the implant region. Vessels were defined by the presence of a lumen surrounded by a complete brown rim of positive hCD31 or α-SMA staining, depending on the quantification. If host erythrocytes were present in the lumens, vessels were considered perfused.

### Measurement of implant volumes

The volumes of model implants fabricated in a manner identical to real implants were evaluated via ultrasound. Instead of injecting constructs into mice, the same volume (500 µL) delivered in vivo was pipetted into the transparent caps of 5 mL Eppendorf Tubes that had been placed in wells of 6-well plates. Samples were then placed in the incubator for 25–30 min at 37 °C to allow complete gelation. To prevent samples from drying, 6 mL of EGM-2 was added to each construct, and constructs were then flipped upside-down and left in the incubator overnight. High frequency ultrasound imaging was employed using the Vevo 770 (VisualSonics Inc., Toronto, Canada) with the same parameters described previously^51^ to image cellular implants and quantify their volumes. The implant volumes were normalized to the volume of the pre-cultured condition, which was found to be close to 500 µL, the expected initial implant volume. Implant volumes for each test condition were averaged over three implants.

### Statistical analysis

Statistical analyses for multiple comparisons for in vitro experiments were performed using one-way ANOVA followed by Tukey’s post hoc test and in vivo experiments were performed using Brown-Forsythe and Welch ANOVA followed by Dunnett’s post hoc test using GraphPad Prism version 8.4.2 for Windows, GraphPad Software, San Diego, California USA, www.graphpad.com. Data are reported as mean ± standard deviation (SD). Values of *p* ≤ 0.05 were considered statistically significant.

## Supplementary information

Supplementary figure S1

## Data Availability

The datasets generated during and/or analyzed during the current study are available from the corresponding author on reasonable request.
