# Supplementary material for: Injectable pre-cultured tissue modules catalyze the formation of extensive functional microvasculature in vivo
Source: Sci Rep. 2020 Sep 23;10:15562. doi: 10.1038/s41598-020-72576-5 (PMC7511337; doi:10.1038/s41598-020-72576-5)
Supplement: Supplementary file 1 — Supplementary figure S1 [file 41598_2020_72576_MOESM1_ESM.pdf]

Injectable Pre-Cultured Tissue Modules Catalyze the Formation of Extensive Functional Microvasculature *in Vivo*

Nicole E. Friend[1,+], Ana Y. Rioja[1,+], Yen P. Kong[1], Jeffrey A. Beamish[2], Xiaowei Hong[1], Julia C. Habif[1], Jonathan R. Bezenah[3], Cheri X. Deng[1], Jan P. Stegemann[1,*], Andrew J. Putnam[1,3,*]

[1]Department of Biomedical Engineering, University of Michigan, Ann Arbor

[2]Division of Nephrology, Department of Internal Medicine, University of Michigan, Ann Arbor

[3]Department of Chemical Engineering, University of Michigan, Ann Arbor

[+]NEF and AYR are co-first authors

[*]JPS and AJP are co-corresponding authors

[*] Corresponding Authors:
Andrew J. Putnam, Ph.D.
Department of Biomedical Engineering
University of Michigan
2204 Lurie Biomedical Engineering Building
1101 Beal Ave. Ann Arbor, MI 48109
Tel: 734-615-1398
Fax: 734-647-4834
Email: putnam@umich.edu

Jan P. Stegemann, Ph.D.
Department of Biomedical Engineering
University of Michigan
2212 Lurie Biomedical Engineering Building
1101 Beal Ave. Ann Arbor, MI 48109
Tel: 734-764-8313
Fax: 734-647-4834
Email: jpsteg@umich.edu

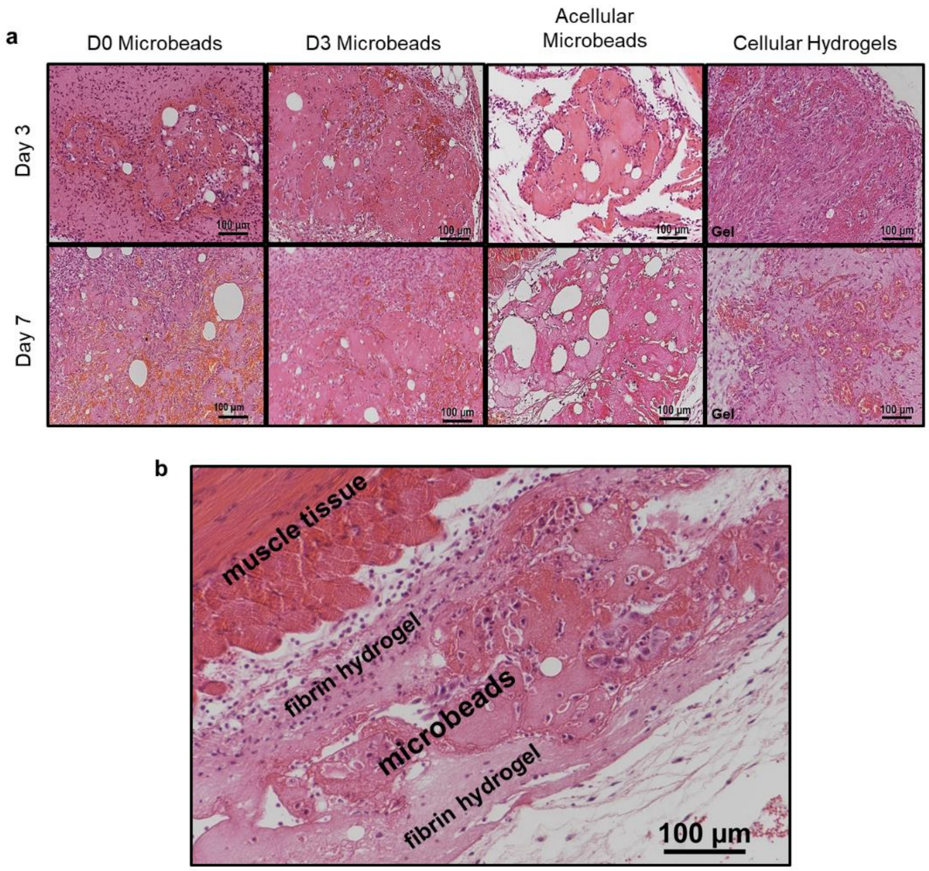

Supplementary Figure 1: Representative images of H&E-stained sections. (a.) Unlabeled version of Figure 3b to allow visualization of histological features in the absence of highlight labels. **(b.)** Representative image at higher magnification showing the histological appearance of different tissue and material types.
